# Effects of Remote Ischemic Preconditioning on Decreasing Troponin Release in Patients Not Taking Sulfonylureas After Cardiac Surgery – A Meta-Analysis

**DOI:** 10.21470/1678-9741-2022-0160

**Published:** 2023

**Authors:** Xiaotong Wang, Shengjue Xiao, Yue Hu, Minjia Guo, Ailin Liu, Chunyan Huan, Tao Xu, Jie Yin, Defeng Pan, Hong Zhu

**Affiliations:** 1 Department of Cardiology, The Affiliated Hospital of Xuzhou Medical University, Xuzhou, Jiangsu, People’s Republic of China; 2 Department of General Practice, The Affiliated Hospital of Xuzhou Medical University, Xuzhou, Jiangsu, People’s Republic of China

**Keywords:** Blood Pressure, Troponin, Cardiac Surgery, Cardiopulmonary Bypass, Sulfonylureas, Meta-Analysis

## Abstract

**Introduction:**

Remote ischemic preconditioning (RIPC) is a new noninvasive myocardial protection strategy that uses blood pressure cuf inflation to simulate transient non-fatal ischemia to protect the myocardium and reduce ischemia-reperfusion injury. Sulfonylureas may mask the effects of RIPC due to their cardioprotec-tive effect. This meta-analysis aimed to evaluate whether RIPC, in the absence of sulfonylureas, reduces troponin release in patients undergoing cardiac surgery.

**Methods:**

We conducted a meta-analysis of randomized controlled clinical trials to determine whether RIPC can reduce postoperative troponin release in cardiac surgery patients undergoing cardiopulmonary bypass without treatment with sulfonylureas. The data were normalized to equivalent units prior to the analysis. A random-effects model was used to provide more conservative estimate of the effects in the presence of known or unknown heterogeneity.

**Results:**

Six studies with a total of 570 participants were included. The analysis showed that troponin release was lower in the RIPC group than in the control group at six hours (test of standardized mean differences = 0, Z=3.64, *P*<0.001) and 48 hours (Z=2.72, *P*=0.007) postoperatively. When the mean of cross-clamping time was > 60 minutes, RIPC reduced troponin release at six hours (Z=2.84, *P*=0.005), 24 hours (Z=2.64, *P*=0.008), and 48 hours (Z=2.87, *P*=0.004) postoperatively.

**Conclusion:**

In cardiac surgery patients who are not taking sulfonylureas, RIPC can reduce troponin release at six and 48 hours postoperatively; hence, RIPC may serve significant benefits in certain cardiac surgery patients.

## INTRODUCTION

Ischemia-reperfusion injury refers to the phenomenon in which reperfusion after ischemia cannot restore the function of a tissue or organ; instead, it aggravates tissue and organ dysfunction and structural damage. Even with the recent advances on understanding the mechanisms that underlie reperfusion injury, the therapeutic results of some mechanisms, such as oxidative stress, Ca^2+^ overload, and anti-inflammatory response, remain unsatisfactory^[^1^]^. In 1986, an experimental study by Murray et al.^[^2^]^ showed that transient non-fatal ischemia-reperfusion attacks on organs or tissues had a strong protective effect against subsequent persistent and fatal ischemia-reperfusion injury, and this phenomenon is known as ischemic preconditioning (IPC). More recent studies^[^3^,^4^]^ have shown that the heart can be protected remotely by applying IPC to an organ, such as the kidney, liver, and intestine, or to tissues that are distant from the heart (*e.g.*, upper or lower limb skeletal muscles); and remote ischemic preconditioning (RIPC) has been proposed to perform this. RIPC is easier to perform in the clinic and it greatly reduces the risk of invasive cardiac injury. Since 2006, trials of RIPC-induced cardiac protection have been conducted clinically^[^5^]^. Subsequently, many clinical trials assessed the cardioprotective effects of RIPC in the context of percutaneous coronary intervention (PCI) and cardiac surgery. Unfortunately, a significant number of randomized controlled trials (RCTs) involving RIPC have been inconclusive about its cardioprotective effects, both based on laboratory indicators and clinical outcomes^[^5^, ^6^, ^7^]^. However, this does not completely deny the potential cardioprotective effect of RIPC, because many confounding factors are present in clinical practice, such as age, drugs, and comorbid diseases, which may ultimately mask the effect of RIPC^[^8^]^. Many researchers have conducted meta-analyses on RIPC studies, and most of them have shown that RIPC reduces postoperative troponin release^[^9^, ^10^, ^11^, ^12^, ^13^]^. However, some of the meta-analysis studies included factors that may interfere with the effect of RIPC; more specifically, studies involving diabetes were not excluded, and this poses a challenge because the drugs that are used to treat diabetes, especially sulfonylureas, also have cardiovascular effects. Sulfonylureas, such as glimepiride and glibenclamide, possibly mask the positive effect of RIPC^[^14^, ^15^, ^16^]^. Sulfonylureas close the adenosine triphosphate-dependent K+ channels of pancreatic beta cells that permit calcium ion inflow, which, in turn, triggers insulin secretion. Considering that these channels are also found in the myocardium, sulfonylureas exhibit several cardioprotective effects, such as preventing action potential shortening during circumscribed myocardial ischemia^[^16^]^. Therefore, in this study, we screened trials that excluded the interference of diabetes drugs, aiming to evaluate the myocardial protective effect of RIPC on patients undergoing cardiac surgery after eliminating the contributing factors of diabetes drugs.

## METHODS

### Search Strategy

The following keywords were searched in the MEDLINE, Excerpta Medica Database (or Embase), Web of Science, Cochrane, and Clinicaltrials databases: “cardiac surgery” or “cardiosurgery” or “coronary artery bypass grafting”, “ischemic preconditioning” or “remote ischemic preconditioning”, “random controlled trial” or “random” or “placebo”.

Registration: This meta-analysis has been registered on PROSPERO (https://www.crd.york.ac.uk/PROSPERO/; ID: CRD42021272239).

### Study Selection

RCTs that compared troponin release after cardiac surgery in adults who had undergone RIPC or not were included. All patients with diabetes that were included should have stopped taking sulfonylureas at least three days before surgery. The exclusion criteria were as follows: trials that involved children, not RIPC of limbs, and trials that failed to identify whether sulfonylureas were used to treat the patients involved. Studies that did not yield any major outcomes were also excluded. The major outcomes of the included trials were levels of cardiac troponin at six, 24, and 48 hours postoperatively.

Two investigators (Hong Zhu and Defeng Pan) independently reviewed the titles, abstracts, and full manuscript texts to determine whether the studies meet the inclusion criteria. Independently and together, the reviewers assessed the risk of bias using the Cochrane Collaboration tool^[^17^]^. All conflicts were resolved through review and discussion.

### Data Extraction

Two authors (Yue Hu and Ailin Liu) independently extracted the relevant data including the baseline characteristics of participants, RIPC protocols, troponin, and other relevant characteristics.

### Statistical Methods

Before the analysis, the data were standardized into equivalent units. Some studies presented data as medians. We used the methods of Luo^[^18^]^ and Wan^[^19^]^ to convert the medians to mean ± standard deviation. We combined the subgroup data using the following formulas:


$$n=n_{1}+n_{2},\;m=\frac{\left(n_{1}m_{1}+n_{2}m_{2}\right)}{n_{1}+n_{2}},SD=\sqrt{\frac{\left(n_{1}-1\right)sd_{1}^{2}+\left(n_{2}-1\right)sd_{2}^{2}+\frac{n_{1}n_{2}}{n_{1}+n_{2}}\left(m_{1}^{2}+m_{2}^{2}-2m_{1}m_{2}\right)}{n_{1}+n_{2}-1}}$$

(Where n=sample size, m=mean, SD=standard deviation)

Data analysis was performed using Review Manager 5.4 (RevMan, The Cochrane Collaboration, Oxford, United Kingdom) and STATA 12.0 (StataCorp, College Station, Texas, United States of America). We calculated the standardized mean differences (SMD) and the corresponding 95% confidence intervals values for continuous variables. A random-effects model was used to provide more conservative estimates of the effects in the presence of known or unknown heterogeneity. Subgroup analyses were performed using the cross-clamping time and different cycles of RIPC. Statistical significance was defined as *P*<0.05.

## RESULTS

### Description of Included Studies

**Fig. 1:**
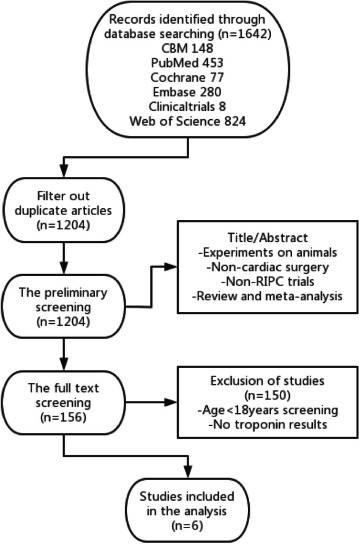
Flow chart of search and selection of studies. CBM=Chinese Biomedical Database; Embase=Excerpta Medica Database; RIPC=remote ischemic preconditioning.

The study selection process is illustrated in Figure 1. We included six studies^[^20^, ^21^, ^22^, ^23^, ^24^, ^25^]^ with a total of 570 participants: 300 in the RIPC group and 270 in the control group. The eligible studies were conducted from 2007 to 2016, and all the studies were conducted in adults. We combined partial data from each experiment separately to facilitate an intuitive presentation (Table 1). All of the patients with diabetes who were taking sulfonylureas stopped treatment at least three days before surgery.

**Table 1:** Basic characteristics of the included studies.

Studies	Total (N)	N		Age (mean±SD)		Male (%)		RIPC protocol		Number of bypass (mean±SD)		Outcome
		RIPC	Control	RIPC	Control	RIPC	Control	Cycle	Limb (N)	RIPC	Control	(T/F)
Hausenloy^[^20^]^, 2007	57	27	30	67+11.8	67±9.4	78%	80%	3*5 min	1	2.81±0.62	2.87±0.68	T
Hong^[^21^]^,2010	130	65	65	65.7+7.5	65.1±9.0	71%	67%	4*5 min	1	2.80±0.90	3.10±0.80	F
Lomivorotov^[^22^]^, 2012	80	40	40	56.8+8.7	58.1±6.4	93%	90%	3*5 min	1	2.60±0.9	2.80±0.70	F
Saxena^[^23^]^,2013	30	15	15	65.1±10.5	68.7±7.8	100%	87%	3*5 min	1	4.40+1.10	4.10±0.50	F
Candilio^[^24^]^,2015	178	89	89	65.0±10.0	66.0±10.0	81%	75%	2*5 min	2	-	-	T
Karami^[^25^]^,2016	95	64	31	59.8+10.3	62.5±10.7	59%	32%	3*5 min	2	2.25+0.44	2.28±0.45	F

Note:T, the results showed that RIPC reduced the release of troponin after operation, and it was statistically significant; and F, the results showed that RIPC did not reduce postoperative troponin release, or decreased troponin release, but it was not statistically significant.

N=Number; RIPC=remote ischemic preconditioning; SD=standard deviation

### Risk of Bias Assessment

**Fig. 2:**
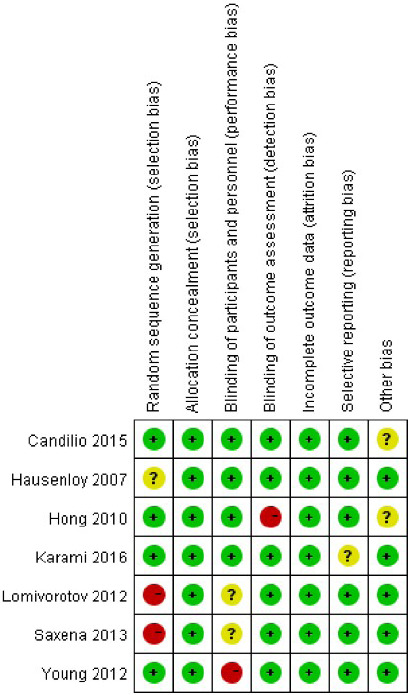
Risk of bias graph. Green=low risk of bias; yellow=unclear risk of bias; red=high risk of bias.

**Fig. 3:**
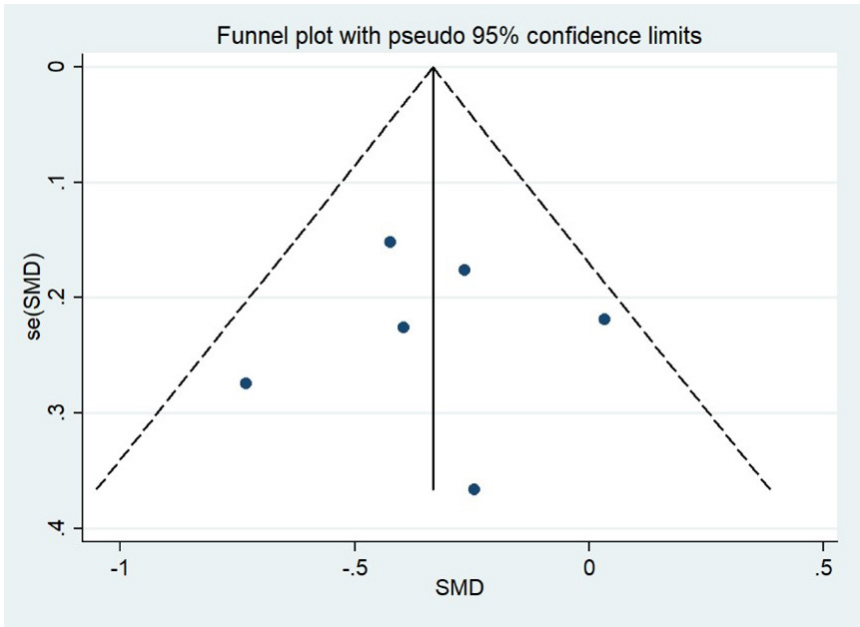
Funnel plot of standard error (se). SMD=standardized mean difference.

The risk of bias summary is shown in Figure 2. Two studies were considered to have a high risk of bias in the random sequence generation because specific randomization was not achieved. Funnel plot analyses (Figure 3) were employed, and symmetry of the funnel plot was observed.

### The Level of Troponin After Surgery

**Fig. 4:**
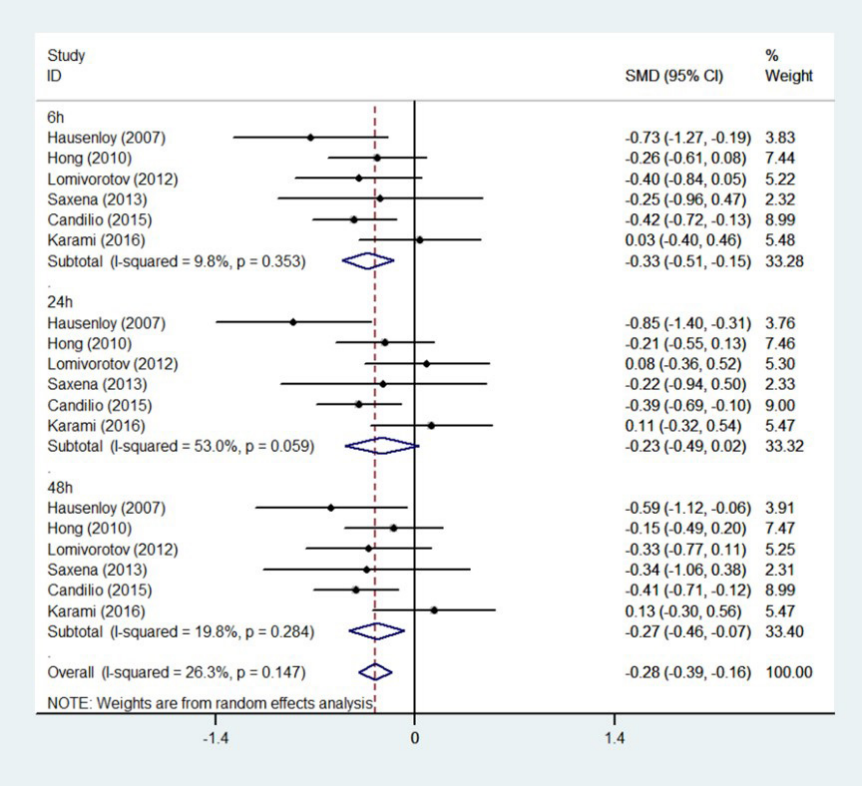
Meta-analysis of standardized mean difference (SMD) and 95% confidence interval (CI) for release of troponin at six, 24, and 48 hours postoperatively. ID=identification.

We analyzed troponin levels at six, 24, and 48 hours postoperatively, and found that at six and 48 hours postoperatively, troponin levels in RIPC group were significantly lower than those in the control group (six hours: test of SMD=0, Z=3.64, *P*<0.001; 48 hours: test of SMD=0, Z=2.72, *P*=0.007). At 24 hours after surgery, the results were not statistically significant (SMD=0, Z=1.78, *P*=0.07) (Figure 4).

### Analysis of RIPC Cycles

**Fig. 5:**
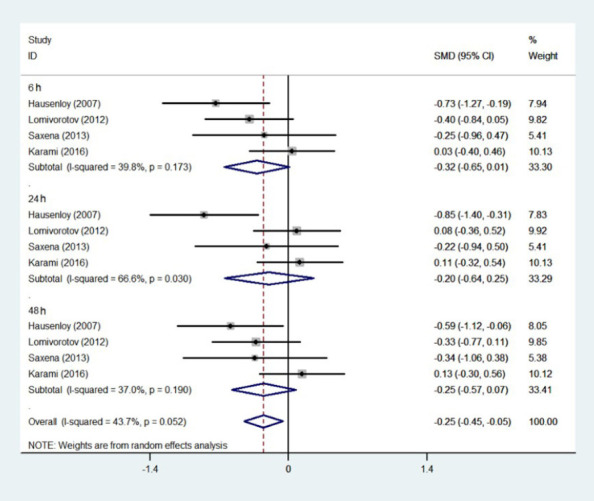
Forest plot of three cycles of remote ischemic preconditioning at six, 24, and 48 hours postoperatively. CI=confidence interval; ID=identification; SMD=standardized mean difference.

Three cycles of RIPC were used in four experiments, and two and four cycles were used in the remaining two experiments. Therefore, we decided to analyze all the experiments using three RIPC cycles. The results showed that RIPC did not reduce troponin release at six, 24, or 48 hours postoperatively in any of the analyzed trials (six hours: test of SMD=0, Z=1.90, *P*=0.058; 24 hours: test of SMD=0, Z=0.86, *P*=0392; 48 hours: test of SMD=0, Z=1.51, *P*=0.130) (Figure 5).

### Subgroup Analysis of Cross-Clamping Time

**Fig. 6:**
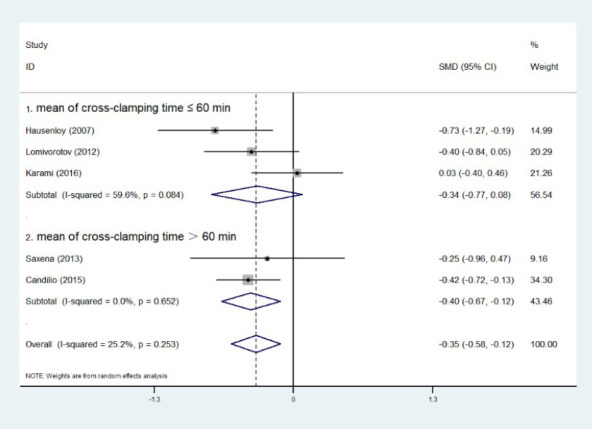
Subgroup analysis of cross-clamping time at six hours postoperatively. CI=confidence interval; ID=identification; SMD=standardized mean difference.

**Fig. 7:**
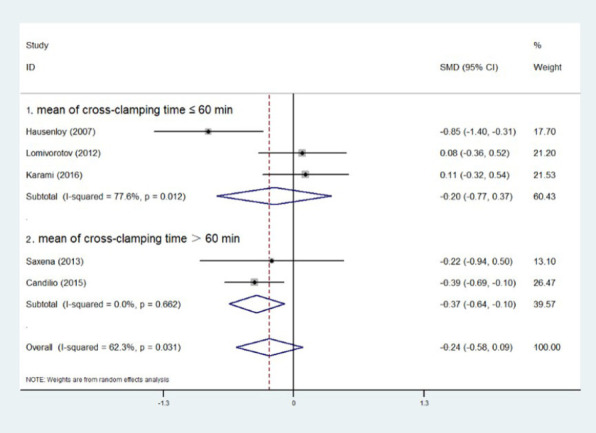
Subgroup analysis of cross-clamping time at 24 hours postoperatively. CI=confidence interval; ID=identification; SMD=standardized mean difference.

**Fig. 8:**
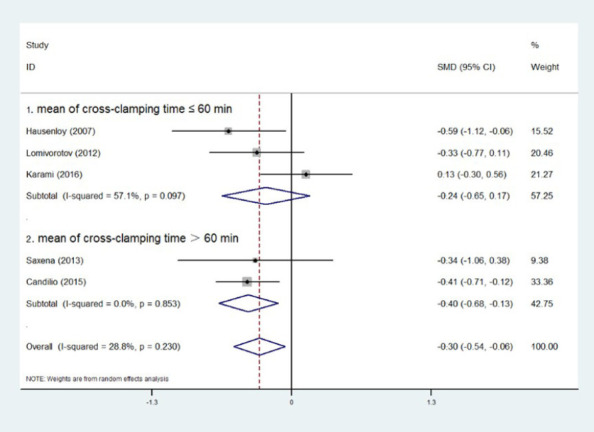
Subgroup analysis of cross-clamping time at 48 hours postoperatively. CI=confidence interval; ID=identification; SMD=standardized mean difference.

Five experiments provided cross-clamping time, and we combined the data of each experiment using the aforementioned formulas. Then, we divided the experiment into two groups based on the mean value of cross-clamping time after merging and whether it was less than, equal to, or greater than 60 minutes. At six hours postoperatively, RIPC showed a significant reduction in troponin levels when the cross-clamping time was > 60 minutes (SMD=0, Z=2.84, *P*=0.005), but not when it was ≤ 60 minutes (SMD=0, Z=1.58, *P*=0.114). The same holds true at 24 hours ([SMD=0, Z=0.67, *P*=0.501] *vs.* [SMD=0, Z=2.64, *P*=0.008]) and 48 hours ([SMD=0, Z=1.15, *P*=0.252] *vs.* [SMD=0, Z=2.87, *P*=0.004]) postoperatively (Figures 6, 7, 8).

## DISCUSSION

Our analysis showed that after the sulfonylurea treatment was stopped, RIPC significantly decreased troponin release in patients undergoing cardiopulmonary bypass at six and 48 hours, but not at 24 hours postoperatively. RIPC is a cardioprotective phenomenon where there are brief periods of ischemia followed by the reperfusion of one organ or tissue that can ultimately confer subsequent protection against ischemia-reperfusion injury in other organs^[^26^]^. RIPC is easier to operate than traditional IPC; its protective mechanism against ischemia-reperfusion is more visible on cardiopulmonary bypass surgery than PCI, since we cannot predict the occurrence of myocardial infarction, but we can master the patterns of myocardial reperfusion after cardiopulmonary bypass. In addition, Carlos et al.^[^27^]^ confirmed the possibility of RIPC preventing anthracycline-induced cardiotoxicity in pig experiments, which expanded the possible clinical applications of RIPC. If conditions permit, we can study RIPC in any predictable myocardial injury to explore its cardioprotective effects.

We chose the three time points (six, 24, and 48 hours postoperatively) for the analysis for the following reasons: the first window of protection immediately follows the stimulus and lasts for 2–3 hours, after which the cardioprotective effect wanes (acute or classic IPC)^[^2^,^28^]^. The second window of protection begins 12–24 hours after the introduction of the stimulus and lasts for 48–72 hours (delayed or late IPC)^[^29^,^30^]^. The results analyzed at 24 hours are inconsistent with previous experimental reports. However, it is worth noting that the results at 24 hours showed a *P*-value of 0.07, which is close to being statistically significant. In addition, analysis of Figure 4 indicates that this finding maybe due to the influence of one or two studies that might have departed from the general trend of decreasing troponin levels post-RIPC. RIPC significantly reduced troponin levels 48 hours after surgery, and this is consistent with the second window of protection, while the results at six hours verified the protective effect of the first window. In a study by Young et al.^[^31^]^, one of the conditions for high-risk surgery was that it requires a longer duration. In cardiopulmonary bypass surgery, the cross-clamping time is equal to the duration of myocardial ischemia; that is, the longer the duration of myocardial ischemia, the higher the risk. Therefore, we conducted a subgroup analysis based on cross-clamping times. The results showed that RIPC had a positive effect on decreasing troponin release when the mean cross-clamping time was > 60 minutes, but the effect was not statistically significant when the cross-clamping time was ≤ 60 minutes. This suggests that RIPC has a better troponin reduction effect in high-risk surgeries, but the length of the cross-clamping time alone does not fully represent the level of risk and it is correlated with the number of bypass grafts performed. Therefore, these results should be interpreted with caution. Currently, researchers tend to use the European System for Cardiac Operative Risk Evaluation (or EuroSCORE) or The Society of Thoracic Surgeons (or STS) mortality risk score to estimate the risk of surgery; we tried to analyze it using these scores, but the number of available studies was very small.

To further study the clinical applications of RIPC, researchers have attempted to assess various extensions of RIPC. The mechanism of RIPC involves the generation of many endogenous factors, and different doses of stimulus may produce different doses of protective factors, that may have a better protective effect. The RIPC stimulation dose can be achieved by increasing the number of RIPC cycles, extending the duration of RIPC, and increasing the number of RIPC in the limbs. Based on the cycles of RIPC subgroup analysis, most studies adopted three cycles; the number used in the previous study^[^32^]^ is recommended. But our analysis results show that the three-loop RIPC is not like an overall analysis of the results, which was statistically significant.

Despite the reduction of troponin release being confirmed only in a few trials and despite most clinical trials showing that RIPC does not significantly improve clinical outcomes, RIPC remains to have a promising role in cardiac protection. For the abovementioned reasons, RIPC is not oficially endorsed or used in clinical practice. Although possible confounding factors have already been discussed, it is impossible to exclude all confounding factors in clinical trials due to individual differences. Some studies suggest that it is uncertain whether RIPC will prove to be helpful or protective in all procedures^[^33^]^. Our study showed that RIPC can reduce the release of troponin post-cardiac surgery, and only after the use of sulfonylureas is discontinued. To some extent, this verified the masking effect of sulfonylureas on the cardioprotective effect of RIPC, and this also considered the different effects of RIPC on different populations (*i.e.*, patients with diabetes). Similarly, the cardioprotective effects of RIPC could be better utilized if other possible confounders are gradually eliminated or diferentiated among different populations. Therefore, the next step is to study the effects of RIPC on different populations. For example, RIPC experiments can be conducted in patients with different risks, comorbidities, and ages. Alternatively, by eliminating possible interfering factors, groups that are more suitable for RIPC may be screened. In addition, the adoption of combination therapy as a multitargeted cardioprotective strategy is also very promising^[^34^]^. RIPC can be combined with other myocardial protection measures to provide significant myocardial protection. In a recent animal study, Claudia Penna et al.^[^35^]^ found that temperature may also affect the effects of RIPC. This provides a new approach for the implementation of RIPC to effectively carry out its cardioprotective effects.

Based on the results of our analysis, we recommend that RIPC be administered to cardiac surgery patients. RIPC may only have significant effects in a subset of the population, but it should still be considered and looked into since no adverse events from it have been reported. At the same time, the applications and effects of RIPC may be limited due to the lack of standardized protocols for RIPC, individual differences in patients, and various confounding factors present in clinical practice.

### Limitations

The main limitation of this study is that only a few references were included. This ultimately resulted in a small sample size, which inevitably increases the risk of bias. This limitation was difficult to avoid due to the following reasons: first, drug withdrawal aggravates other concomitant diseases; second, some factors that influence RIPC are difficult to exclude; and third, there are only a few relevant trials available in the literature. Additionally, different troponin types have different effects on patient prognosis, however we were not able to perform further subgroup analyses based on each type. Moreover, the adopted RIPC protocols in the trials were different from one another (*i.e.*, not standardized), and factors, such as different RIPC cycles and intervention limbs, may serve as potential sources of risk.

## CONCLUSION

After the discontinuation of sulfonylureas, RIPC can reduce troponin release at six and 48 hours postoperatively in cardiac surgery patients, which confirmed the cardioprotective effect of RIPC. We support the application of RIPC in cardiac surgery and suggest that subsequent clinical trials assess the cardioprotective effects of RIPC in various specific or special populations.
